# Efficacy of Colchicine in Reducing Cardiovascular Events in Patients with Acute Coronary Syndrome: A Systematic Review

**DOI:** 10.7759/cureus.86424

**Published:** 2025-06-20

**Authors:** Mayuri Quishpe, Mirella Sanunga, Jhon Ochoa, Jennifer Cunuhay, Nicolás Haro, Darlyn Paredes, Wilson Cabrera, Jesus Endara-Mina

**Affiliations:** 1 Research, Escuela Superior Politécnica de Chimborazo, Riobamba, ECU; 2 Research, San Andrés Hospital, Santo Domingo, ECU; 3 Ecuavolcan Research Group, Pontificia Universidad Católica del Ecuador, Quito, ECU

**Keywords:** acute coronary syndrome, adverse effects, cardiovascular events, colchicine, efficacy

## Abstract

Acute coronary syndrome (ACS) remains one of the leading causes of cardiovascular morbidity and mortality, largely due to the high incidence of recurrent adverse events. Colchicine, a traditional anti-inflammatory drug primarily used for the treatment of gout, has emerged as a potential therapeutic option in cardiovascular care due to its ability to stabilize atherosclerotic plaques. This systematic review aimed to evaluate the efficacy of colchicine in reducing cardiovascular events and inflammatory biomarkers in patients with ACS. Eight studies published between 2013 and 2024 were included, selected through a comprehensive search of databases such as PubMed, ScienceDirect, Cochrane Library, and SciELO, as well as manual searches in scientific journals like the New England Journal of Medicine and via the Google Scholar search engine. Eligibility criteria included randomized, double-blind, placebo-controlled clinical trials assessing patients diagnosed with ACS. A total of 18,759 patients were analyzed, 9,369 in the intervention group (colchicine 0.5 mg/day plus standard treatment) and 9,390 in the placebo group, with follow-up periods ranging from three months to three years. Both primary outcomes (major cardiovascular events including myocardial infarction, stroke, and recurrent ischemia) and secondary outcomes (reduction of inflammatory biomarkers such as hs-CRP and IL-6 and incidence of adverse effects) were assessed. The results demonstrated that colchicine was associated with a significant reduction in hs-CRP and IL-6 levels, as well as a lower incidence of adverse cardiovascular events, particularly myocardial infarction and recurrent myocardial ischemia. In conclusion, colchicine may represent a promising adjunctive therapeutic alternative for patients with ACS, particularly due to its anti-inflammatory effects and potential role in secondary prevention. However, further studies with greater methodological consistency and long-term follow-up are required to confirm these benefits.

## Introduction and background

Cardiovascular diseases (CVDs) remain the leading cause of morbidity and mortality worldwide [1]. In 2019, the World Health Organization (WHO) reported that CVDs accounted for approximately 17.9 million deaths, representing 32% of all global deaths, disproportionately affecting populations in low- and middle-income countries [2]. Over 85% of these deaths are attributed to myocardial infarction and stroke, with coronary artery disease being the primary cause of premature death across most populations. These figures underscore the magnitude of the problem and the urgent need for more effective preventive and therapeutic strategies [3].

Myocardial infarction, along with unstable angina, is a hallmark manifestation of acute coronary syndrome (ACS). It is estimated that over seven million people are diagnosed with ACS worldwide each year and approximately 5% of ACS patients die before hospital discharge [4,5]. The high rate of recurrent cardiovascular events in ACS patients highlights the need for therapeutic approaches that address not only the acute phase of the disease but also the prevention of long-term complications [3,6].

Despite significant advances in the management of ACS, particularly early revascularization and antithrombotic therapies, current treatment options remain suboptimal. Standard therapies fail to eliminate the residual risk of recurrent cardiovascular events and tend to oversimplify the complex nature of ischemic risk. Moreover, many of these treatments are costly and often inaccessible in resource-limited settings. This context has renewed attention toward the inflammatory component of atherosclerosis, now recognized as a key factor in the development of unstable atherosclerotic plaques, particularly during acute ischemic events such as myocardial infarction [6].

Inflammation plays a central role in the pathophysiology of atherosclerosis and its complications. In this context, the NLRP3 (NOD-, LRR-, and pyrin domain-containing protein 3) inflammasome is an intracellular multiprotein complex that acts as a key danger signal sensor. It consists of the NLRP3 sensor, the ASC (apoptosis-associated speck-like protein containing a CARD) adaptor protein, and procaspase-1, which becomes active caspase-1 upon activation. This complex recognizes various stimuli, such as cholesterol crystals, extracellular ATP, lipopolysaccharides, and damage-associated molecular patterns (DAMPs), all of which are commonly present in the inflammatory and stressful microenvironment of atherosclerotic plaques [7,8].

The activation of the NLRP3 inflammasome occurs in two well-defined steps. The first, known as "priming", is triggered by signals that induce the increased transcriptional expression of NLRP3 and pro-IL-1β via NF-κB activation. The second step, or activation, involves the assembly of the inflammasome complex in response to specific danger signals, such as potassium efflux, increased reactive oxygen species, or the presence of cholesterol crystals [8].

Once activated, the NLRP3 inflammasome promotes the activation of caspase-1, which plays a crucial role in processing and maturing the pro-inflammatory cytokines IL-1β and IL-18, which are subsequently released into the extracellular space. These cytokines contribute to amplifying the local and systemic inflammatory response, promoting leukocyte recruitment and tissue damage, thereby increasing plaque instability and rupture, ultimately triggering acute ischemic events such as myocardial infarction [8]. Furthermore, inflammasome activation can induce a form of programmed cell death known as pyroptosis, which releases additional pro-inflammatory mediators and perpetuates the inflammatory cycle.

Colchicine inhibits NLRP3 inflammasome activation by interfering with microtubule cytoskeletal dynamics required for inflammasome assembly in macrophages and monocytes. This process reduces caspase-1 activation and the subsequent release of IL-1β and IL-18, thus modulating vascular inflammation. This specific anti-inflammatory mechanism makes colchicine a promising therapeutic candidate for stabilizing atherosclerotic plaques and reducing recurrent cardiovascular events, with a well-characterized pharmacological profile and low cost [9].

Recent trials such as COVERT [10] and COLCOT [11] have demonstrated that colchicine is associated with a significant reduction in recurrent cardiovascular events in patients with ACS, including cardiovascular death, myocardial infarction, and stroke, suggesting that colchicine may effectively enhance current standard-of-care regimens for the secondary prevention of ACS. However, further research is needed to evaluate its long-term benefits and potential adverse events, particularly gastrointestinal toxicity. Given the limitations of existing therapies, the discovery of the inflammatory basis of atherosclerosis has captured clinical attention, as inflammation is now acknowledged to play a central role in plaque instability during acute episodes such as myocardial infarction [9].

Based on this background, a key question arises: What is the effectiveness of colchicine as an anti-inflammatory strategy for reducing cardiovascular events in patients with ACS? Therefore, this study aims to evaluate colchicine by considering both its clinical benefits and the incidence of adverse effects, in order to provide evidence that clarifies its therapeutic role in this patient population.

## Review

Methodology

Research Aim 

The objective of this systematic review was to assess the efficacy of colchicine, compared to placebo, in reducing cardiovascular events in patients diagnosed with ACS. The research question was structured using the PICO framework, where the population comprised adult patients with a confirmed diagnosis of ACS (including ST-segment elevation myocardial infarction, non-ST-segment elevation myocardial infarction, and unstable angina), the intervention consisted of the daily administration of colchicine at a dose of 0.5 mg as an adjunct to standard therapy, the comparator was placebo plus standard therapy, and the primary outcome was the reduction of major adverse cardiovascular events (MACE). 

This study is registered in the International Prospective Register of Systematic Reviews (PROSPERO) system under the identification number CRD420251026729. The registered protocol was modified once, specifically to expand the age range of included participants by removing the initial upper limit of 75 years. This change was justified by evidence found in several of the selected studies, which included populations with a mean age exceeding that threshold.

Search Strategy 

A comprehensive and systematic search strategy was implemented across various electronic bibliographic databases, including PubMed/MEDLINE, Cochrane Library, ScienceDirect, and SciELO. Additionally, a manual search was conducted in the New England Journal of Medicine, and Google Scholar was used as a complementary source.

The search strategy was designed using Medical Subject Headings (MeSH) terms and relevant synonyms, combined with Boolean operators. The following search string was applied: ("Colchicine" OR "colchicine") AND ("Acute Coronary Syndrome" OR "acute coronary syndrome" OR "ACS") AND ("Cardiovascular Events" OR "myocardial infarction" OR "stroke" OR "major adverse cardiovascular events" OR "MACE") AND ("Randomized Controlled Trial" OR "RCT" OR "clinical trial"). The search was restricted to articles published between January 2017 and December 2024, in English or Spanish. Articles in other languages were excluded due to operational limitations in technical translation. The Preferred Reporting Items for Systematic Reviews and Meta-Analyses (PRISMA) 2020 guidelines [12] were followed for study identification and selection. All references were manually reviewed to remove duplicates.

Selection Criteria 

Selected studies had to be randomized controlled trials (RCTs), double-blinded, and placebo-controlled, conducted in adult patients with a confirmed diagnosis of ACS. Additionally, studies had to report at least one of the following outcomes: incidence of MACE (e.g., myocardial infarction, stroke, or cardiovascular death), levels of inflammatory biomarkers (hs-CRP or IL-6), or adverse events associated with the intervention. Observational studies, narrative or systematic reviews, editorials, and letters to the editor were excluded, as were studies using different colchicine dosages or with a follow-up period shorter than three months.

Data Extraction and Management 

Data extraction was performed using a standardized form adapted from the Cochrane Consumers and Communication Review Group template. The form was pilot-tested in two studies to ensure clarity and applicability, thereby reducing potential errors during data collection.

Extracted data included information on authorship, year of publication, journal, country and study setting, total and group-specific sample sizes, type of intervention, duration of follow-up, primary and secondary outcomes, reported adverse events, and ethical considerations such as institutional ethics committee approval and informed consent. Two independent reviewers (M.Q. and M.S.) assessed the relevance of titles and abstracts. Discrepancies were resolved by consensus or by consulting a third reviewer (J.E.).

Analysis and Synthesis of Data 

Due to substantial clinical and methodological heterogeneity across the included studies, primarily concerning follow-up duration, outcome definitions, and population characteristics, a meta-analysis was not feasible. Instead, a qualitative narrative synthesis was conducted, grouping results based on outcome type (cardiovascular events, inflammatory biomarkers, adverse events) and follow-up duration (less than or greater than 12 months). Two investigators (J.O. and J.C.) independently extracted the data, and differences were resolved through discussion or arbitration by a third reviewer (J.E.).

Quality Assessment 

The methodological quality of the included RCTs was assessed using the JADAD scale [5] and the GRADE approach to evaluate the certainty of evidence. Each study was assigned a level of evidence and a quality rating (high, moderate, low, or very low). Only studies scoring ≥3 on the JADAD scale and with at least moderate certainty according to GRADE were included in the final synthesis.

Evaluation of the Study 

As part of the evaluation process, it was confirmed that all included studies had been approved by an institutional ethics committee and had obtained informed consent from participants. Additionally, potential risks of selection, performance, detection, attrition, and reporting biases were analyzed in accordance with GRADE criteria. To ensure methodological rigor, a checklist based on the PRISMA 2020 guidelines was applied to evaluate compliance with quality standards in study selection, evaluation, and synthesis. Only evidence meeting minimum ethical and methodological criteria was included in the final synthesis, thereby enhancing the validity and reliability of the results.

Results

**Figure 1:**
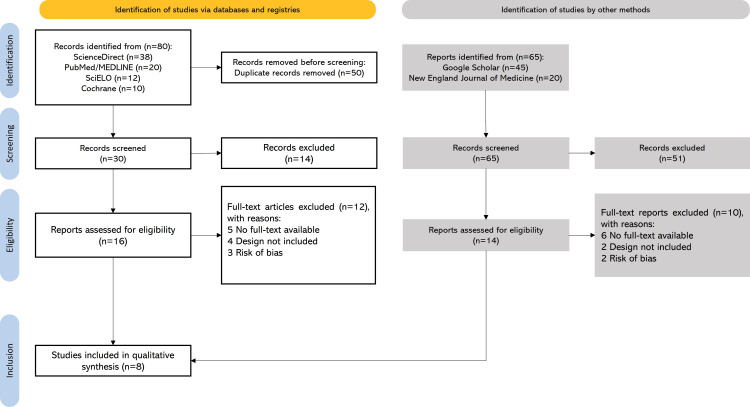
PRISMA flow diagram of study screening and selection PRISMA: Preferred Reporting Items for Systematic Reviews and Meta-Analyses

Using predefined search strategies and specific eligibility criteria, a total of 145 articles were initially identified: 80 through electronic databases and 65 through other complementary search methods. A total of 30 full-text articles were assessed for eligibility according to the inclusion criteria (Figure 1). Ultimately, eight RCTs, published in either English or Spanish, were included in the qualitative synthesis.

Synthesis of Results

The included studies were RCTs, double-blinded and placebo-controlled, all employing a quantitative design, conducted between 2017 and 2025 in European and Asian countries. Collectively, these studies analyzed a total of 18,759 participants, 9,369 were assigned to the colchicine group (0.5 mg/day) and 9,390 to the placebo group, with both arms receiving standard therapy for ACS. Follow-up periods ranged from three months to three years.

Regarding the outcomes assessed, six studies evaluated the incidence of MACE (such as myocardial infarction, stroke, and cardiovascular death), two studies focused on the reduction of inflammatory biomarkers such as hs-CRP and IL-6, and four reported on adverse events, particularly mild gastrointestinal effects.

The methodological quality of the studies was assessed using the JADAD scale, which assigns a maximum score of 5 points. Five studies scored ≥4, indicating high quality, while three studies scored 3, corresponding to moderate quality. The GRADE tool was also used to assess the certainty of the evidence for the primary outcomes. Domains evaluated included risk of bias, inconsistency, imprecision, indirectness, and publication bias. Overall, the certainty of the evidence ranged from moderate to high for most clinically relevant outcomes.

Key data from each study, including author, year of publication, country, title, study design, sample size, follow-up duration, type of intervention, evaluated variables, and main results, are presented in Table 1.

**Table 1:** Randomized clinical trials used in the systematic review ACS: acute coronary syndrome; CI: confidence interval; FCT: fibrous cap thickness; GMR: geometric mean ratio; HR: hazard ratio; hs-CRP: high-sensitivity C-reactive protein; IL-6: interleukin-6; IQR: interquartile range; IS: infarct size; LV: left ventricular; MACE: major adverse cardiovascular events; MPO: myeloperoxidase; OR: odds ratio

No.	Author, year, and country	Title of the clinical trial	Design	Sample; follow-up time	Intervention	Variables	Results	Scale of GRADE; certainty	Scale of JADAD
1	Opstal et al., 2021, Netherlands and Australia [13]	Colchicine in Patients With Chronic Coronary Artery Disease in Relation to Prior Acute Coronary Syndrome	Randomized, double-blind, placebo-controlled clinical trial	Sample: 5,522 patients with ACS. Colchicine: 2762 patients. Placebo: 2760 patients. Without previous ACS: n=864. Recent ACS (6-24 months): n=1,479. Remote ACS (2-7 years): n=1,582. Very remote ACS (>7 years): n=1,597. Follow-up: median of 28.6 months. Most patients were around 2.5 years old	Colchicine 0.5 mg once daily or placebo. Without prior ACS: Colchicine 0.5 mg/day vs. placebo. Recent ACS: Colchicine 0.5 mg/day vs. placebo. Remote ACS: Colchicine 0.5 mg/day vs. placebo. Very remote ACS: Colchicine 0.5 mg/day vs. placebo	MACE: Spontaneous myocardial infarction. Ischemic stroke. Ischemia-induced coronary revascularization	Colchicine: Patients without previous ACS: The effects of colchicine compared with placebo on the risk of cardiovascular death, spontaneous myocardial infarction, ischemic stroke, or ischemic coronary revascularization were as follows: incidence: 2.8 vs. 3.4 events per 100 person-years; HR: 0.81; and 95% CI: 0.52-1.27). Patients with previous ACS: Incidence: 2.4 vs. 3.6 events per 100 person-years; HR: 0.67; 95% CI: 0.54-0.82) (P for interaction=0.43). Placebo: Patients without previous ACS: Recent ACS (incidence: 2.4 versus 3.3 events per 100 person-years; HR: 0,75; 95% CI: 0.51-1.10). Distant ACS (incidence: 1.8 vs. 3.2 events per 100 person-years; HR: 0.55; 95% CI: 0.37-0.82). Very remote ACS (incidence: 3.0 vs. 4.3 events per 100 person-years; HR: 0.70; 95% CI: 0.51-0.96; P for interaction=0.59)	High	4/5 (adequate quality)
2	Akrami et al., 2021, Iran [14]	Effects of Colchicine on Major Adverse Cardiac Events in the 6-Month Period After Acute Coronary Syndrome Occurrence: A Randomized, Placebo-Control Trial	Randomized, double-blind, placebo-controlled clinical trial	Sample: 249 patients. Colchicine: 120 patients. Placebo: 129 patients. Follow-up: 6 months	Colchicine 0.5 mg/day plus standard treatment vs. placebo plus standard treatment	MACE. Mortality. Gastrointestinal adverse effects	Significantly lower rates of MACE in favor of colchicine (6.7% vs. 21.7%; HR 1.64; 95% CI 1.31-2.05; analysis was statistically significant at p=0.001). In the group treated with colchicine, 4 deaths occurred (3.3%), while in the placebo group. 2 deaths were recorded (1.6%); the statistical analysis did not show significance, with p=0.35. Fifteen patients (12.5%) in the colchicine group experienced gastrointestinal symptoms, with diarrhea being the most common adverse effect. On the other hand, in the placebo group, only three patients (2.5%) experienced gastrointestinal symptoms. This difference was statistically significant at p=0.002	High	4/5 (adequate quality)
3	Tong et al., 2020, Australia [15]	Colchicine in Patients With Acute Coronary Syndrome	Randomized, double-blind, placebo-controlled multicenter clinical trial	Sample: 795 patients. Colchicine: 396 patients. Placebo: 399 patients. Follow-up: 12 months	Intervention group: Colchicine 0.5 mg twice daily for 1 month and then 0.5 mg/day for 11 months. Control group: Placebo plus standard therapy	MACE included ACS, urgent revascularization, and non-cardioembolic ischemic stroke. Mortality. Gastrointestinal adverse effects (diarrhea, flatulence, abdominal pain)	MACE: Colchicine group: 24 events (6.1%). Placebo group: 38 events (9.5%). HR: 0.65 (95% CI: 0.38-1.09). This difference was statistically significant at p=0.09. Colchicine: 11 events (2.8%). Placebo: 20 events (5%). HR: 0.56 (95% CI: 0.27-1.18). This difference was not statistically significant at p=0.13. Colchicine: 3 events (0.8%). Placebo: 12 events (3%). HR: 0.26 (95% CI: 0.07-0.92). This difference was statistically significant at p=0.037. Colchicine: 8 deaths (2%). Placebo: 1 death (0.3%). HR: 8.20 (95% CI: 1.03-65.61). This difference was statistically significant at p=0.018. Five of the eight deaths in the colchicine group were non-cardiovascular, related to infections (sepsis). Colchicine: 91 patients (23%). Placebo: 83 patients (20.8%). This difference was not statistically significant, with p=0.46	Moderate	4/5 (adequate quality)
4	Yu et al., 2024, China [16]	Effect of Colchicine on Coronary Plaque Stability in Acute Coronary Syndrome as Assessed by Optical Coherence Tomography: The COLOCT Randomized Clinical Trial	Randomized, double-blind, placebo-controlled clinical trial	Sample: 128 patients. Colchicine: 64 patients. Placebo: 64 patients. Follow-up: 12 months	Intervention group: Colchicine 0.5 mg/day plus standard therapy. Control group: Placebo plus standard therapy	FCT. Reduction in mean lipid arc. Macrophage infiltration. Inflammatory biomarkers: hs-CRP, IL-6, and MPO. Adverse effects: Gastrointestinal	Colchicine: 34.2 (95% CI: 9.7-58.6]; this difference was statistically significant at p=0.006. Placebo: 51.9 (95% CI: 32.8-71.0). Colchicine: reduction of -35.7 (95% CI: -40.5 to -30.8). Placebo: reduction of -25.2 (95% CI: -30.6 to -19.9). Difference in change: -10.5 (95% CI: -17.7 to -3.4). This difference was statistically significant at p=0.004. Colchicine: reduction of -14.0 (95% CI: -18.0 to -10.0). Placebo: -8.9% reduction (95% CI: -13.3% to -4.6%). Change difference: -6% (95% CI: -11.8% to -0.2%). This difference was statistically significant at p=0.044. hs-CRP (GMR), 0.6% (95% CI: 0.4-1%) vs. 0.3% (95% CI: 0.2-0.5%); difference: 0.5% (95% CI: 0.3-1%). This difference was statistically significant (p=0.046) for the colchicine group. IL-6 (GMR: 0.8 (95% CI: 0.6-1.1) vs. 0.5 (95% CI: 0.4-0.7); difference: 0.6 (95% CI: 0.4-0.9). This difference was statistically significant (p=0.025) for the colchicine group. MPO (GMR, 1.0 (95% CI: 0.8-1.2) vs. 0.8 (95% CI: 0.700.9); difference: 0.8 (95% CI: 0.6-1.0). This difference was statistically significant (p=0.047) for the colchicine group. The frequencies of gastrointestinal symptoms with placebo versus colchicine were 6.3% versus 9.4% (p=0.744); for diarrhea, 1.6% versus 4.7% (p=0.619); for nausea, 1.6% versus 1.6% (p=1.000); and for flatulence, 3.1% versus 3.1% (p=1.000) not statistically significant	High	5/5 (adequate quality)
5	Montarello et al., 2021, Australia [17]	Assessing the Impact of Colchicine on Coronary Plaque Phenotype After Myocardial Infarction With Optical Coherence Tomography: Rationale and Design of the COCOMO-ACS Study	Randomized, double-blind, placebo-controlled clinical trial	Sample: 64 patients. Colchicine: 32 patients. Placebo: 32 patients. Follow-up: 18 months	Intervention group: Colchicine 0.5 mg/day plus standard therapy. Control group: Placebo plus standard therapy	Minimum fibrous cap thickness. Inflammatory biomarker hs-CRP	Colchicine: mean increase of 97% (95% CI: 77-117%). Placebo: mean increase of 65% (95% CI: 40-90%). Difference: 32% (95% CI: 10-54%). This difference was statistically significant at p=0.02. Colchicine: 50% reduction. Placebo: 20% reduction. This difference was statistically significant at p=0.04	High	5/5 (adequate quality)
6	Tardif et al., 2019, International [18]	Efficacy and Safety of Low-Dose Colchicine After Myocardial Infarction	Randomized, double-blind, placebo-controlled clinical trial	Sample: 4,745 patients. Colchicine 2,366 patients. Placebo 2,379 patients. Follow-up time: Median 22.6 months	Intervention group: Colchicine 0.5 mg/day plus standard therapy. Control group: Placebo plus standard therapy	Composite outcome of cardiovascular death, resuscitated cardiac arrest, myocardial infarction, stroke, and unstable angina requiring hospitalization. Myocardial infarction. Stroke. Unstable angina	Colchicine: 187 events (7.1%). Placebo: 264 events (11%). HR: 0.77 (95% CI: 0.61-0.96). This difference was statistically significant at p=0.02. HR 0.84 (95% CI: 0.61-1.17). P=0.05 (not significant). HR 0.26 (95% CI: 0.10-0.70; p=0.008) (statistically significant). HR 0.50 (95% CI: 0.31-0.81; p=0.005) (statistically significant)	High	5/5 (adequate quality)
7	Jolly et al. 2024, Multicentric (14 countries) [19]	Colchicine in Acute Myocardial Infarction	Randomized, multicenter, prospective, placebo-controlled clinical trial, 2×2 factorial design	Sample: 7,062 patients. Colchicine 3,528 patients. Placebo 3,534 patients. Follow-up: 3 years	Intervention group: Colchicine 0.5 mg/day plus standard therapy. Control group: Placebo plus standard therapy	Primary clinical events: composite of cardiovascular death, recurrent myocardial infarction, stroke, unscheduled coronary revascularization. Inflammatory biomarker: C-reactive protein at 3 months	Colchicine: 322 events (9.1%). Placebo: 327 events (9.3%). HR: 0.99 (95% CI: 0.85-1.16); p=0.93 (not significant). Colchicine: 322/3528 (9.1%). Placebo: 327/3534 (9.3%). HR: 0.99 (95% CI: 0.85-1.16); p=0.93 (not statistically significant)	Moderate	4/5 (adequate quality)
8	Mewton et al. 2021, France [20]	Effect of Colchicine on Myocardial Injury in Acute Myocardial Infarction	Randomized, double-blind, multicenter, placebo-controlled clinical trial	Sample: 192 patients. Colchicine 101 patients. Placebo 91 patients. Follow-up: 3 months	Intervention group: A loading dose of 2 mg oral colchicine, followed by 0.5 mg twice daily for 5 days. Control group: Placebo plus a loading dose and then twice daily for 5 days	IS (g of the left ventricular mass) at 5 days. IS (g of the left ventricular mass) at 3 months. Relative left ventricular remodeling (% change in end-diastolic volume). LV thrombus at 5 days. MACE at 3 months. Gastrointestinal adverse effects (diarrhea, nausea)	Colchicine group: Median 26 g (IQR: 16-44 g). Placebo group: Median 28.4 g (IQR: 14-40 g). There were no significant differences (p=0.87). Colchicine group: Median 17.2 g (IQR: 10.5-27.8 g). Placebo group: Median 18.4 g (IQR: 10-26.5 g). There were no significant differences (p=0.97). Colchicine group: +2.4% (IQR: -8.3% to +11.1%). Placebo group: -1.1% (IQR: -8% to +9.9%). There were no significant differences (p=0.48). Colchicine group: 18 patients (22.2%). Placebo group: 6 patients (7.4%). OR: 4.05 (95% CI: 1.46-12.67). This difference was statistically significant at p=0.01. There were no significant differences between groups (p=0.66). Colchicine group: 33 patients (34.4%). Placebo group: 9 patients (10.1%). P=0.0001 (significantly increased with colchicine)	Moderate	5/5 (adequate quality)

Discussion

Following a rigorous process of literature collection and analysis, evidence was found supporting the use of colchicine in reducing cardiovascular events in patients with ACS. However, the findings are mixed, as some studies reported positive outcomes, while others did not demonstrate statistically significant differences. In total, eight studies were identified which, collectively, support the hypothesis proposed in the introduction of this review, although the heterogeneity of the results must be taken into account.

Among the reviewed studies, colchicine stands out as a traditional anti-inflammatory agent used in the treatment of gout that has emerged as a potential therapeutic option in the cardiovascular field, particularly for the secondary prevention of coronary artery disease [21,22].

Colchicine exerts its anti-inflammatory effect by inhibiting the NLRP3 inflammasome, a multiprotein complex found in immune cells such as neutrophils, monocytes, and eosinophils. This inflammasome plays a pivotal role in the production of pro-inflammatory cytokines, primarily IL-1β and IL-18, which are involved in vascular inflammation and the development of atherosclerotic plaques [23,24]. By blocking the activation of the NLRP3 inflammasome, colchicine reduces the production of IL-1β and IL-18, thereby decreasing vascular inflammation and modulating platelet-leukocyte interactions. This contributes to slowing the progression and preventing the destabilization of atherosclerotic plaques [23,25].

The inhibition of these cytokines is particularly relevant in the context of ACS, as IL-1β and IL-18 play a central role in plaque inflammation and instability. IL-1β promotes endothelial inflammation and facilitates the migration of inflammatory cells into the plaque, leading to extracellular matrix degradation and smooth muscle cell apoptosis, which weakens plaque integrity. In turn, IL-18 amplifies the inflammatory response by stimulating the production of additional cytokines and activating immune cells. This chronic inflammatory state can result in a thin fibrous cap, increasing the plaque's vulnerability to rupture. Plaque rupture exposes its contents to the bloodstream, often triggering thrombus formation and, consequently, acute ischemic events such as myocardial infarction or stroke. Therefore, by inhibiting IL-1β and IL-18, colchicine contributes to plaque stabilization and reduces the risk of cardiovascular complications in ACS patients.

Colchicine is administered orally, in either solid or liquid form. It is rapidly absorbed in the gastrointestinal tract, metabolized in the liver, binds to albumin at approximately 40%, and is excreted mainly as metabolites in feces (around 80%), with 10-20% eliminated via urine. As a result, patients receiving colchicine require ongoing monitoring, particularly those with hepatic dysfunction or renal impairment [26,27]. From an economic standpoint, low-dose colchicine has been recognized as a cost-effective strategy for the secondary prevention of CVD [11].

Consequently, several studies have evaluated the efficacy of colchicine in patients with chronic coronary disease and ACS. For example, the LoDoCo2 trial conducted by Opstal et al. in the Netherlands and Australia, which included 5,522 patients with chronic coronary disease, demonstrated that a daily dose of 0.5 mg of colchicine significantly reduced the incidence of MACE, regardless of prior ACS history. Specifically, a consistent reduction was observed in the composite outcome of cardiovascular death, spontaneous myocardial infarction, ischemic stroke, and ischemia-driven coronary revascularization across subgroups, including patients with no prior ACS and those with recent, remote, or very remote ACS events [13]. Similarly, the study by Nidorf et al. indicated that the risk of cardiovascular events was significantly lower among patients receiving 0.5 mg of colchicine daily compared to those receiving a placebo [28].

The COLCOT trial [18], with a robust sample size and a median follow-up of 22.6 months, showed a significant reduction in the composite endpoint (HR 0.77; 95% CI: 0.61-0.96; p=0.02), with additional benefits in the reduction of stroke and unstable angina. Comparable findings were reported in the study by Akrami et al. [14], which demonstrated a significant reduction in MACE at six months (6.7% vs. 21.7%; p=0.001). However, in the more recent trial by Jolly et al. [19], the largest to date, no statistically significant differences were observed in primary events (HR: 0.99; 95% CI: 0.85-1.16; p=0.93), raising questions about the universal effectiveness of colchicine across all post-ACS clinical scenarios.

A meta-analysis conducted in Argentina by Masson et al., which included seven studies and enrolled 5,966 patients in the colchicine group and 5,948 in the control group, found that colchicine use was significantly associated with a reduced risk of acute myocardial infarction (AMI) (OR: 0.76; 95% CI: 0.62-0.92; I²=15%) [29]. A reduction in stroke incidence was also observed; however, there was no significant impact on cardiovascular mortality, as also reported in the study by Akrami et al., where mortality did not reach statistical significance (p=0.35) [14].

Studies such as the CANTOS trial by Ridker et al., although utilizing canakinumab instead of colchicine, confirmed that lipid-independent inflammation reduction can lower cardiovascular event rates, thereby validating the anti-inflammatory therapeutic approach [30]. Based on these findings, Montarello et al. and Yu et al. used optical coherence tomography to demonstrate increased fibrous cap thickness and reduced macrophage infiltration in patients with ACS treated with colchicine, further supporting the hypothesis of vulnerable plaque stabilization [16,17].

Nonetheless, in the study by Mewton et al., early colchicine administration in AMI patients did not result in significant differences in infarct size or ventricular remodeling after three months, as neither infarct volume nor echocardiographic parameters of remodeling differed between groups. These results suggest that colchicine may not substantially alter acute myocardial injury but may exert its main benefits during the subacute or chronic phase of residual inflammation, reinforcing its value in secondary prevention rather than direct myocardial protection [20].

From a biochemical perspective, recent scientific evidence supports that elevated levels of fibrinogen, IL-6, hs-CRP, and galectin-3 are significantly associated with increased cardiovascular risk [31]. Therefore, these inflammatory biomarkers are emerging as potential tools for the risk stratification and early prediction of cardiovascular events, although their prognostic utility presents certain limitations [32].

In this context, the effects of colchicine on inflammatory biomarkers have been consistently demonstrated in secondary prevention. This is supported by a recent meta-analysis by Alam et al., which evaluated colchicine's impact on hs-CRP levels in ACS patients. The analysis revealed that colchicine therapy was significantly associated with lower hs-CRP levels (MD -1.59; 95% CI: -2.40 to -0.79; p=0.0001), indicating reduced systemic inflammation and, consequently, a lower incidence of future major adverse clinical events [33]. Another meta-analysis conducted in Australia by Razavi et al., which included seven clinical trials, found that colchicine administration was associated with a significant reduction in serum CRP levels (OR: 0.67; 95% CI: 0.46-0.98; p=0.04; I²=46%) and a 70% reduction in stroke risk in the colchicine-treated group. However, no significant difference was found in myocardial infarction incidence between the colchicine and control groups (OR: 0.89; 95% CI: 0.67-1.17; p=0.39; I²=0%) [34]. This discrepancy may be explained by differences in patient populations, timing of treatment initiation, and duration of follow-up.

On the other hand, it is essential to thoroughly assess colchicine's safety profile. Four studies, notably the COPS trial by Tong et al., reported a higher incidence of adverse effects, particularly gastrointestinal symptoms such as diarrhea and nausea in 23% of cases [15], potentially compromising long-term treatment adherence. Similarly, a meta-analysis of eight trials comparing colchicine to placebo suggested that colchicine may increase the incidence of diarrhea in primary prevention, although the evidence remains highly uncertain (57/309 (18.4%) vs. 22/296 (7.4%); RR 3.99; 95% CI: 1.44-11.06; Tau²=0.94; I²=51%; eight studies, 605 participants; very low certainty of evidence) [35]. It is worth noting that severe adverse events were rare and that most symptoms were self-limited, as confirmed by a safety analysis by Deftereos et al., which indicated that although rare, serious infections may occur in vulnerable patients with chronic diseases due to mild colchicine-induced immunosuppression [22].

Ultimately, both the clinical trials and meta-analyses reviewed provide robust evidence supporting the efficacy of low-dose colchicine as part of standard secondary preventive therapy. The observed risk reduction is comparable to that of other secondary prevention strategies, such as antithrombotic therapy [26]. Given that colchicine has demonstrated effectiveness in reducing major ischemic events, such as myocardial infarction and stroke, a potential reduction in cardiovascular mortality may be inferred [36]. However, while a lower incidence of cardiovascular deaths was observed in colchicine-treated groups, this difference did not reach statistical significance. Notably, all included trials were conducted in populations with established CVD, but most reported deaths were attributable to non-cardiovascular causes, limiting the ability to detect a significant effect on overall mortality.

Limitations

This systematic review has several limitations that must be considered when interpreting the results. First, although multiple RCTs and meta-analyses were included, heterogeneity among the studies in terms of design, population characteristics, duration of follow-up, and outcome definitions may affect the generalizability of the results. For example, differences in the timing of colchicine administration following an acute coronary event may influence its anti-inflammatory effects and clinical efficacy. Furthermore, the inclusion of both patients with stable coronary artery disease and those with ACS may introduce confounding factors.

Second, despite efforts to include only high-quality studies, the possibility of publication bias cannot be entirely ruled out, as positive results tend to be more frequently published than negative or inconclusive findings. Moreover, some included studies reported open-label designs or had limited sample sizes, which could compromise the internal validity and increase the risk of bias in estimating treatment effects.

Third, while this review incorporated mechanistic evidence on inflammatory biomarkers and plaque stabilization through advanced imaging techniques, such as optical coherence tomography, the number of studies in this field remains limited, and these findings should be interpreted with caution.

Finally, most of the included studies were conducted in high-income countries with robust healthcare systems, which may limit the applicability of the results to populations in low- and middle-income countries, where access to medications and monitoring may be restricted [37].

## Conclusions

Colchicine, an anti-inflammatory agent with a long-established safety profile, has emerged as a promising therapeutic option for the secondary prevention of cardiovascular events in patients with ACS. Its mechanism of action, based on the inhibition of the NLRP3 inflammasome and reduction of key inflammatory cytokines such as IL-1β and IL-18, supports its potential to stabilize atherosclerotic plaques and reduce the incidence of recurrent ischemic events.

The evidence analyzed in this review, including randomized clinical trials and meta-analyses, suggests that low-dose colchicine (0.5 mg/day) is associated with a significant reduction in major adverse cardiovascular events, including myocardial infarction and stroke, particularly in patients with elevated inflammatory markers or residual inflammatory risk. Additionally, its impact on inflammatory biomarkers, such as hs-CRP, provides further support for its role in modulating cardiovascular risk.

However, the absence of a consistent reduction in cardiovascular mortality across studies, the variability in adverse effect profiles, particularly gastrointestinal intolerance, and the heterogeneity in study designs underscore the need for additional large-scale, high-quality clinical trials. These future studies should aim to determine the optimal timing, duration, and patient selection criteria for colchicine therapy, as well as to assess its cost-effectiveness and safety in diverse clinical and geographic settings. Colchicine represents a low-cost, well-tolerated, and potentially effective strategy for reducing residual inflammatory risk in patients with ACS. Its incorporation into current treatment algorithms should be based on individualized risk-benefit assessments and ongoing clinical research.

## References

[1] Radovanovic M, Jankovic J, Mandic-Rajcevic S, Dumic I, Hanna RD, Nordstrom CW (2023). Ideal cardiovascular health and risk of cardiovascular events or mortality: a systematic review and meta-analysis of prospective studies. J Clin Med.

[2] World Health Organization (2025). Cardiovascular diseases. WHO.

[3] Nelson K, Fuster V, Ridker PM (2023). Low-dose colchicine for secondary prevention of coronary artery disease: JACC review topic of the week. J Am Coll Cardiol.

[4] Timmis A, Kazakiewicz D, Townsend N, Huculeci R, Aboyans V, Vardas P (2023). Global epidemiology of acute coronary syndromes. Nat Rev Cardiol.

[5] Wang H, Zulikaier T, Yumaierjiang B, Lyu S, He P (2025). Platelet-to-lymphocyte ratio efficiency in predicting major adverse cardiovascular events after percutaneous coronary intervention in acute coronary syndromes: a meta-analysis. Rev Cardiovasc Med.

[6] Melendo-Viu M, Marchán-Lopez Á, Guarch CJ (2023). A systematic review and meta-analysis of randomized controlled trials evaluating pharmacologic therapies for acute and recurrent pericarditis. Trends Cardiovasc Med.

[7] Kodi T, Sankhe R, Gopinathan A, Nandakumar K, Kishore A (2024). New insights on NLRP3 inflammasome: mechanisms of activation, inhibition, and epigenetic regulation. J Neuroimmune Pharmacol.

[8] Robertson S, Martínez GJ, Payet CA, Barraclough JY, Celermajer DS, Bursill C, Patel S (2016). Colchicine therapy in acute coronary syndrome patients acts on caspase-1 to suppress NLRP3 inflammasome monocyte activation. Clin Sci (Lond).

[9] Vaidya K, Tucker B, Kurup R (2021). Colchicine inhibits neutrophil extracellular trap formation in patients with acute coronary syndrome after percutaneous coronary intervention. J Am Heart Assoc.

[10] Bouleti C, Viscogliosi S, Bresson D (2024). Colchicine in acute myocardial infarction: cardiovascular events at 1-year follow up. Open Heart.

[11] Samuel M, Tardif JC, Khairy P (2021). Cost-effectiveness of low-dose colchicine after myocardial infarction in the Colchicine Cardiovascular Outcomes Trial (COLCOT). Eur Heart J Qual Care Clin Outcomes.

[12] Page MJ, McKenzie JE, Bossuyt PM (2021). The PRISMA 2020 statement: an updated guideline for reporting systematic reviews. BMJ.

[13] Opstal TS, Fiolet AT, van Broekhoven A (2021). Colchicine in patients with chronic coronary disease in relation to prior acute coronary syndrome. J Am Coll Cardiol.

[14] Akrami M, Izadpanah P, Bazrafshan M, Hatamipour U, Nouraein N, Drissi HB, Manafi A (2021). Effects of colchicine on major adverse cardiac events in next 6-month period after acute coronary syndrome occurrence; a randomized placebo-control trial. BMC Cardiovasc Disord.

[15] Tong DC, Quinn S, Nasis A (2020). Colchicine in patients with acute coronary syndrome: the Australian COPS randomized clinical trial. Circulation.

[16] Yu M, Yang Y, Dong SL (2024). Effect of colchicine on coronary plaque stability in acute coronary syndrome as assessed by optical coherence tomography: the COLOCT randomized clinical trial. Circulation.

[17] Montarello NJ, Singh K, Sinhal A (2022). Assessing the impact of colchicine on coronary plaque phenotype after myocardial infarction with optical coherence tomography: rationale and design of the COCOMO-ACS study. Cardiovasc Drugs Ther.

[18] Tardif JC, Kouz S, Waters DD (2019). Efficacy and safety of low-dose colchicine after myocardial infarction. N Engl J Med.

[19] Jolly SS, d'Entremont MA, Lee SF (2025). Colchicine in acute myocardial infarction. N Engl J Med.

[20] Mewton N, Roubille F, Bresson D (2021). Effect of colchicine on myocardial injury in acute myocardial infarction. Circulation.

[21] Coven D (2020). Acute coronary syndrome treatment & management. https://emedicine.medscape.com/article/1910735-treatment?form=fpf.

[22] Deftereos SG, Beerkens FJ, Shah B (2022). Colchicine in cardiovascular disease: in-depth review. Circulation.

[23] Grebe A, Hoss F, Latz E (2018). NLRP3 inflammasome and the IL-1 pathway in atherosclerosis. Circ Res.

[24] Casula M, Andreis A, Avondo S, Vaira MP, Imazio M (2022). Colchicine for cardiovascular medicine: a systematic review and meta-analysis. Future Cardiol.

[25] Roubille F, Tardif JC (2020). Colchicine for secondary cardiovascular prevention in coronary disease. Circulation.

[26] Martí-Carvajal AJ, De Sanctis JB, Hidalgo R (2022). Colchicine for the primary prevention of cardiovascular events. Cochrane Database Syst Rev.

[27] Imai S, Momo K, Kashiwagi H, Miyai T, Sugawara M, Takekuma Y (2020). Prescription of colchicine with other dangerous concomitant medications: a nation-wide survey using the Japanese Claims Database. Biol Pharm Bull.

[28] Nidorf SM, Fiolet AT, Mosterd A (2020). Colchicine in patients with chronic coronary disease. N Engl J Med.

[29] Masson W, Lobo M, Lavalle-Cobo A, Molinero G (2021). Can colchicine prevent acute myocardial infarction? Systematic review and meta-analysis. Rev Argent Cardiol.

[30] Ridker PM, Everett BM, Thuren T (2017). Antiinflammatory therapy with canakinumab for atherosclerotic disease. N Engl J Med.

[31] Liu Y, Guan S, Xu H, Zhang N, Huang M, Liu Z (2023). Inflammation biomarkers are associated with the incidence of cardiovascular disease: a meta-analysis. Front Cardiovasc Med.

[32] Netala VR, Hou T, Wang Y, Zhang Z, Teertam SK (2025). Cardiovascular biomarkers: tools for precision diagnosis and prognosis. Int J Mol Sci.

[33] Alam M, Kontopantelis E, Mamas MA, Savinova OV, Jhaveri A, Siddiqui E, Jhamnani S (2023). Meta-analysis of the effect of colchicine on C-reactive protein in patients with acute and chronic coronary syndromes. Coron Artery Dis.

[34] Razavi E, Ramezani A, Kazemi A, Attar A (2022). Effect of treatment with colchicine after acute coronary syndrome on major cardiovascular events: a systematic review and meta-analysis of clinical trials. Cardiovasc Ther.

[35] Chen T, Liu G, Yu B (2023). A meta-analysis evaluating efficacy and safety of colchicine for prevention of major cardiovascular events in patients with coronary artery disease. Clin Res Cardiol.

[36] Kelly P, Lemmens R, Weimar C (2024). Long-term colchicine for the prevention of vascular recurrent events in non-cardioembolic stroke (CONVINCE): a randomised controlled trial. Lancet.

[37] Kirkner R (2024). New favorable evidence for the use of colchicine in the treatment of DCVA. https://portugues.medscape.com/verartigo/6511076?form=fpf.

